# Rethinking policy perspectives on childhood stunting: time to formulate a structural and multifactorial strategy

**DOI:** 10.1111/mcn.12254

**Published:** 2016-05-17

**Authors:** S V Subramanian, Iván Mejía‐Guevara, Aditi Krishna

**Affiliations:** ^1^ Department of Social and Behavioral Sciences Harvard T.H. Chan School of Public Health Boston Massachusetts USA; ^2^ Child Health Evaluative Sciences and the Centre for Global Child Health SickKids Research Institute, Hospital for Sick Children Toronto ON Canada

**Keywords:** stunting, undernutrition, childhood, cognition, economic growth, support‐led strategy, social determinants, upstream interventions, multifactorial, India

## Abstract

Stunting and chronic undernutrition among children in South Asia remain a major unresolved global health issue. There are compelling intrinsic and moral reasons to ensure that children attain their optimal growth potential facilitated via promotion of healthy living conditions. Investments in efforts to ensure that children's growth is not faltered also have substantial instrumental benefits in terms of cognitive and economic development. Using the case of India, we critique three prevailing approaches to reducing undernutrition among children: an over‐reliance on macroeconomic growth as a potent policy instrument, a disproportionate focus on interpreting undernutrition as a demand‐side problem and an over‐reliance on unintegrated single‐factorial (one at a time) approaches to policy and research. Using existing evidence, we develop a case for support‐led policy approach with a focus on integrated and structural factors to addressing the problem of undernutrition among children in India.

**Key messages**

- Eliminating child undernutrition is important from an intrinsic perspective and offers considerable instrumental benefits to individual and society.
- Evidence suggests that an exclusive reliance on a growth‐mediated strategy to eliminate stunting needs to be reconsidered, suggesting the need for a substantial support‐led strategy.
- Interpreting and addressing undernutrition as a demand‐side problem with proximal single‐factorial interventions is futile.
- There is an urgent need to develop interventions that address the broader structural and upstream causes of child undernutrition.

## Introduction

**Figure 1:**
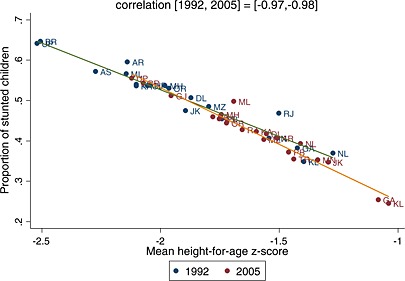
Correlation between mean height‐for‐age *z*‐scores (HAZ) and prevalence (expressed as proportion) of children who were stunted in 1992–1993 and 2005–2006. Source: Authors' calculations using data from National Family Health Survey (NFHS)‐1 and NFHS‐3. State abbreviations: AS, Assam; BR, Bihar; GA, Goa; GJ, Gujarat; HR, Haryana; JK, Jammu and Kashmir; KA, Karnataka; KL, Kerala; MH, Maharashtra; MN, Manipur; ML, Meghalaya; MZ, Mizoram; NL, Nagaland; OR, Odisha; PB, Punjab; RJ, Rajasthan; UP, Uttar Pradesh; DL, New Delhi; AR, Arunachal Pradesh.

If children under the age of 5 years from countries as diverse as Brazil, Ghana, India, Norway, Oman and the United States are exposed to optimal environmental conditions, a distributional plot of their normalized height, given their age, tends to follow a normal distribution (WHO MGRS 2006a). Our expectation would then be that under such optimal environmental conditions, only 2.5% of the children should fall below two standard deviations (SD) from median height, leading to a state commonly referred to as ‘stunting’, a well‐established marker of chronic undernutrition (Corsi *et al.* 2011). However, in South Asia, the percentage of the children who are stunted is nearly 38% (UNICEF 2015). India, with a prevalence of 48% stunting, alone accounts for over 90% of the regional burden, and a third of the global burden (UNICEF 2015). Prioritizing and developing preventive as well as curative public health strategies to address this ‘high‐risk’ (stunted) group cannot be more obvious. It is, however, equally important to note that the extremely high prevalence of stunting suggests a broader underlying phenomenon of chronic undernutrition that affects all children in South Asia. The near perfect positive correlation between mean ‘height‐for‐age’, expressed as *z*‐scores, and the proportion of children who are stunted (Fig. 1) suggests that a ‘population approach’ (Rose 1992) might be more appropriate to address the problem of chronic undernutrition among children in South Asia.

In this essay, we critique two perspectives that are commonplace in the policy, as well as scientific, discourses on addressing chronic undernutrition in childhood. The first relates to the disproportionately high reliance on macroeconomic growth as an instrument to reduce chronic undernutrition among children (Ravallion 1990; Pritchett & Summers 1997; Smith & Haddad 2002). While it is certainly plausible that increases in macroeconomic growth can lead to reductions in chronic undernutrition, we scrutinize the degree of empirical support for the potency of macroeconomic growth to reduce chronic undernutrition. The second prevailing idea relates to the disproportionate focus on ‘demand‐side’ interventions with an aim to addressing ‘proximal’ risk factors (Bhutta *et al.* 2013), leaving the broader structural and supply‐side determinants largely untouched (WHO 2008). Indeed, dramatic improvements in child health in the early parts of the 19th century in much of the industrialized nations were largely facilitated by changes in supply‐side structural determinants (Cutler & Miller 2005; Semba 2008).

In this perspective, we focus on India as an example for developing our arguments given that Indian children experience the greatest share of the regional as well as the global burden on chronic undernutrition (UNICEF 2015). For brevity, we use the term undernutrition or stunting to reflect experiences of chronic undernutrition during childhood. We start with a brief overview on the underlying public health significance of stunting. This is followed by a critique of the ‘growth‐mediated’ strategy to reducing child undernutrition. In the two subsequent sections, we describe and discuss concerns related to the prevailing framing of undernutrition largely as a demand‐side issue problem along with a focus on one risk factor at a time failing to see the connections between different risk factors. In the concluding section, we argue for an urgent need to bolster a concerted support‐led strategy focused on improving the structural and multifactorial determinants of child undernutrition in India.

## Why should we care about stunting?

Expanding human freedom to live the kind of lives that people have a reason to value – as articulated by Nobel Laureate Professor Amartya Sen – provides a compelling framework to consider public health issues within the context of development (Sen 1999). Ensuring that a child reaches his or her underlying potential with regard to their physical and mental development can be seen as a freedom that should be valued for its own sake. An *intrinsic* perspective provides moral reasoning to considering undernutrition within a human rights framework. The 2013 National Food Security Act (Ministry of Law and Justice 2013), which aims to reduce child undernutrition through ensuring the elimination of one of the key causative factors (i.e. access to food), can be seen as an example of valuing the promotion of child development as an end in itself. As is often the case, promotion of such basic human capabilities and freedoms (e.g. ensuring conditions that facilitate optimal child development) invariably tends to bring about tremendous *instrumental* benefits leading to the formation of what is referred to as ‘human capital’ through their indirect role in influencing economic production and social change (Sen 1997). We briefly summarize the positive micro‐ and macro‐socioeconomic gains that can be realized through substantial reductions in stunting.

### Childhood stunting, cognitive development and schooling

Stunting has biological implications for brain development and neurological functioning that translate into cognitive impairment. Experiencing disadvantage early in life has significant implications for cognitive and social development by changing brain architecture and neurochemistry because neural plasticity is greatest during this time (Shonkoff & Phillips 2000; Knudsen *et al.* 2006). During this period of rapid change and development, brains adapt to the environmental conditions (Knudsen *et al.* 2006) with lasting changes in the prefrontal cortex affecting attention and memory as well as by reductions in dendritic density in the hippocampus that impair memory formation and consolidation (Hoddinott *et al.* 2013a). Other effects of undernutrition include diminished myelination of axon fibres, which reduces the speed at which neurological signals are transmitted (Hoddinott *et al.* 2013a). There is also significant evidence that many of these changes persist over the life course (Knudsen *et al.* 2006; Hoddinott *et al.* 2013a).

Several studies have found support for these biological and neurological implications of stunting, translating broadly into robust associations between stunting and cognitive impairment. In a recent systematic review and meta‐analysis of 68 observational (cross‐sectional and prospective) studies on linear growth and cognitive development in 29 low‐income and middle‐income countries, it was shown that children with lower height‐for‐age *z*‐scores (HAZ) in the first 2 years had poorer cognitive outcomes (Sudfeld *et al.* 2015). In children ages 2 years or younger, one SD increase in HAZ was associated with a 0.24 SD [95% confidence interval (CI) 0.14–0.33] increase in cognitive ability (Sudfeld *et al*. 2015). Effects sizes among children over age 2 years were about a third the size (Sudfeld *et al.* 2015), suggesting that nutritional status early in life may be critical for cognitive development. Using longitudinal data from prospective birth cohorts in Brazil, Guatemala, India, Philippines and South Africa, Adair and colleagues (2013) found that faster linear growth was associated with higher schooling attainment with a one SD increase in conditional height at age 2 years associated with a half‐year increase in schooling (Adair *et al.* 2013).

The associations observed in observational studies between stunting and adult educational outcomes have also been validated in studies using experimental designs. For instance, in Guatemala, nutritional supplementation in early childhood led to improvements in adult educational outcomes with a higher educational attainment of 1.2 grades for women and 0.25‐SD increase in reading comprehension and non‐verbal cognitive ability tests for both women and men (Maluccio *et al.* 2009). In short, nutritional deprivation in early childhood, as reflected in growth faltering in height, negatively affects children's cognitive development.

### Childhood stunting and economic production

Early health investments allow individuals to gain even greater health stock later in life, and thereby influencing economic outcomes such as earnings potential and productivity in adulthood. There are two pathways through which a connection between childhood stunting and adult economic production could be conceptualized, measured largely through increased wages and productivity (Horton & Steckel 2011).

The first is a direct pathway whereby adults with better nutritional status, thus in better health (Perkins *et al*., forthcoming), are physically able to work and are likely to have higher output than those in poor nutritional status, while the second is an indirect pathway whereby stunted children, due to comorbidities and poor health status and lack of optimal cognitive development, are less likely to attain the same levels of schooling as healthy children and thereby have lower adult productivity and wages through lower levels of human capital (Horton & Steckel 2011).

While disentangling the two pathways is challenging, what appears to be clear is that stunting is correlated with lower adult wages or earning potential (Deolalikar 1988; Behrman & Deolalikar 1989; Strauss & Thomas 1996; Thomas & Strauss 1997; Schultz 2002, 2003; Hanushek & Wößmann 2008; Behrman *et al.* 2010; Dewey & Begum 2011; Hoddinott *et al.* 2013b).

## Limits to ‘trickle‐down’ economics

Despite the intrinsic importance of ensuring equitable access to opportunities and conditions that matter for child development, as well as the well‐established instrumental benefits to individuals, society and economy, current policy perspectives have failed to recognize the seriousness of the problem of undernutrition. For instance, the current Vice Chairman of India's National Institution for Transforming India Aayog, the country's premier policy commission that works with states to promote economic development, Dr Arvind Panagariya, has gone to the extent of referring to the presence of child undernutrition in India as a ‘myth’ (Panagariya 2012). While it is relatively easy to counter and dismiss such a scientifically ignorant view (Table 1), it is the broader and long‐standing prevailing belief that increased macroeconomic growth will automatically lead to alleviation of several of India's social and health challenges, including undernutrition, which merits critical scrutiny.

**Table 1:** Summary of the arguments made by Professor Arvind Panagariya (2012) and counter arguments

Panagariya's arguments	Counter‐argument
1. The WHO Multi Growth Reference Study (WHO MGRS) cannot be applied to India. He questioned whether the WHO‐MGRS sample is an adequate reference for India – or other developing countries, in terms of geographical, cultural, socioeconomic and genetic backgrounds.	MGRS was designed to study growth among healthy children in five sites from Brazil, Ghana, India, Norway, Oman and the United States to develop growth standards rather than references such as in the National Center for Health Statistics/WHO 1977 growth curves (Wable 2013).
	One of the MGRS sites used was based in New Delhi (WHO MGRS Reference Study Group 2006).
	Of the total variation in child in length/height only, 3% of the variation was between‐sites and is nearly twenty times lower than variability between individuals (WHO MGRS Reference Study Group 2006).
	It is also well established that children in India who have experienced healthy conditions have been able to grow according to international norms (Agarwal *et al*. 1991).
2. Indians are genetically short.	If true, there should be little variation in height‐for‐age within India.
	Yet, large socioeconomic differences *within* India. For instance, stunting prevalence among the richest and poorest quartile is 27% and 40%, respectively (Fig. 3).
3. Mortality is coming down, so by extension, children must be growing optimally.	There is a misleading perception created under the label of “enigma” or “puzzle” with the suggestion that child/infant mortality indicators are somehow better even when prevalence of stunting is high, sparking unnecessary conjectures. The evidence stands directly in contrast to such perceptions. Within India, states that have lower levels of stunting also have lower levels of child/infant mortality (Fig. 2a); a strong and positive relationship between stunting and child/infant mortality is also observed among low and middle‐income countries (Fig. 2b).
	Notwithstanding the strong correlation between child/infant mortality and stunting rates, it is obvious that factors influencing child survival on one hand, and an optimal child growth on other can be different (Gillespie 2013).
	Data on mortality are *estimates*; not observed, and based on the self‐reports by respondents (usually mothers) on their entire life and death history of every child born. It remains unclear if the estimates of mortality are under or over‐biased.
	On the other hand, anthropometry is objectively measured.
4. Comprehensive medical exams should be used instead of anthropometry to measure nutritional status. Malnutrition needs to be distinctly characterized as either protein energy malnutrition or micronutrient deficiency.	While there are other measures of nutritional status, anthropometric measures are well established and are reliable (Corsi *et al.* 2011), and often the different measures have high correlation.
	Classification of malnutrition into two discrete categories of protein energy malnutrition and micronutrient deficiency is unfounded in scientific knowledge (Gillespie 2013; Gupta *et al.* 2013; Wable 2013).
	Focusing on protein, energy and micronutrient intake ignores the broader context of malnutrition in India that is rooted in social, economic, political and environmental conditions (Gillespie 2013; Gupta *et al.* 2013; Wable 2013; Coffey *et al.* 2014).

WHO, World Health Organization.

*Note:* Also see Gupta *et al*. (2013) for a detailed critique of Panagariya (2012).

It is not an exaggeration to state that in countries with low levels of per capita income, such as India, increasing the rate of economic growth is often justified as a key policy instrument to improving population health and nutrition (Pritchett & Summers, 1997; Smith & Haddad 2002). There are several reasons why increased economic growth could potentially lead to reductions in undernutrition. At the macro level, economic growth could lead to improvements in child nutrition through the following: (1) creating new industries and thereby expanding employment opportunities leading to increases in standard of living; (2) enabling greater allocation of resources towards social welfare programmes; and (3) increasing spending on health programmes (Subramanyam *et al.* 2011). These macro‐level changes, in theory, could translate into raising household income and reducing income‐poverty, educational attainment and food security, which then affect nutritional status for children (Subramanyam *et al.* 2011).

India did experience a period of sustained economic growth during the nineties as well as early part of the 2000s, with growth rates greater than 7% between 1994 and 1997 and about 8% or greater rate in 2004 and 2005 (Basu & Maertens 2007). Yet, over the two decades spanning the mid‐1990s to the mid‐2000s – the period when Indian economy was rapidly growing – the prevalence of stunting among children in India declined from 57% (1992–1993) to 39% (2013–2014), a reduction of 1.5% percentage points per year. Furthermore, there were considerable socioeconomic disparities in declines in stunting, with the richest quintile of Indian households experiencing a decline of 14 percentage points between 1992–1993 and 2013–2014, while the poorest quintile experienced a 13 percentage points decline over 21 years (Fig. 3). While such a casual perusal should hardly form the basis for evidence‐based public policy, rigorous analysis using well‐established econometric techniques also questions the proposition that increased economic growth might be a potent policy instrument to reduce undernutrition among children in India.

**Figure 2:**
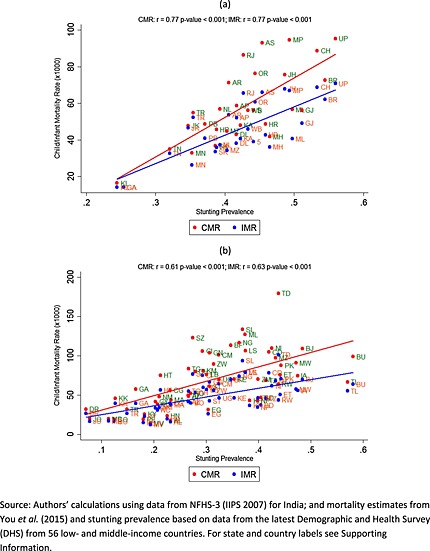
Association between stunting prevalence and child mortality rate (CMR)/infant mortality rate (IMR) among (a) Indian states and (b) 56 low‐ and middle‐income countries

In a comprehensive study that examined the role of state‐level economic growth in explaining the decline in child undernutrition in India, the correlation between growth and undernutrition was close to zero; an Indian rupees (INR) 5000 increase in state per capita economic growth was associated with an odds ratio of 1.02 (95% CI: 0.99–1.05) for stunting (Subramanyam *et al.* 2011). Indeed, although an ecological association was found between *levels* of childhood stunting and *levels* of state per capita economic growth [*R* ^2^(1992–1993, 2005–2006) = (0.06, 0.12)], it was not statistically significant (Fig. 4a), and analyses of associations between *change* in per capita economic growth and *change* in childhood stunting revealed no significant results (Fig. 4b). Null associations were observed for both the lowest and highest quintile wealth groups (Fig. 4b). We conducted sensitivity analysis by approximating state‐specific domestic product by wealth quintile and examining the association between change in stunting prevalence and change in economic growth in the richest quintile groups (fourth and fifth groups), confirming our initial findings of no association (see Table A1 and details of the estimation in the Supporting Information).

**Figure 3:**
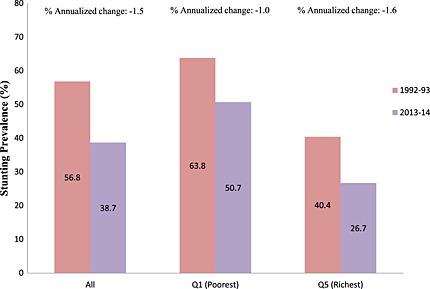
Prevalence of childhood stunting (overall, poorest and richest household wealth quintiles in 1992‐93 (< 48 months) and 2013–2014 (< 60 months). Source: Authors' calculations using data from National Family Health Survey (NFHS)‐1 and Rapid Survey on Children (RSOC) 2013–2014.

The evidence from India that macroeconomic growth did not contribute to the reduction in child undernutrition has also been corroborated at the global level. In an analysis of 121 Demographic and Health Surveys in 36 low‐and middle‐income countries, associations between country‐level macroeconomic growth and reductions in childhood stunting ranged from zero to quantitatively very weak and were extremely robust to a wide range of econometric specifications (Vollmer *et al.* 2014; Vollmer *et al.* 2015).

### Why macroeconomic growth did not matter

Even in the face of empirical evidence, it is challenging to alter long‐held positions about the supposed potential of macroeconomic growth to improve health outcomes. For instance, in response to the aforementioned study, researchers continued to argue that the role of economic growth in reducing child undernutrition should not be dismissed (Alderman *et al.* 2014), even though little evidence was provided to support this statement (Vollmer *et al.* 2015). At the same time, it is perfectly plausible why increases in economic growth did not translate to reductions in child stunting in India.

First, average economic growth hides the huge imbalance in growth across different sectors; put simply, growth was not inclusive, and not all sectors of the population or regions of the country participated in the economic growth in the same manner (Kohli 2006a, 2006b). Indeed, the association between macroeconomic growth and changes in aggregate poverty at the state level was at best modest (Joe *et al.* 2016).

Second, given the fact that not all populations (and especially those involved in low‐performing economic sectors) participated in the growth, it then becomes necessary for the national and state governments to engage in redistributing income *post*‐growth to those excluded population groups in order for the excluded groups to experience improvements in standard of living. There is again no evidence of any such income re‐distribution (direct or indirect) to compensate for the non‐inclusive growth; on the contrary, income inequality appears to have gone up during this period (Dev & Ravi 2007). There is also little evidence that macroeconomic growth led to re‐distribution in an indirect manner such that public development expenditures that matter for social well‐being and health increased (Joe *et al.* 2016).

Third, economic growth did not change any of the proximal risk factors of stunting, such as deficiencies in iron, iodine, vitamin A, zinc or suboptimal breastfeeding (Black *et al.* 2008). Building on Aguayo *et al.* (2014), we constructed a Child Nutrition Score (CNS) using National Family Health Survey (NFHS)‐1 and NFHS‐3 and found no statistical association between the change in the state‐level CNS and the change in state‐level economic growth between 1993 and 2005 (Fig. 5).

**Figure 4:**
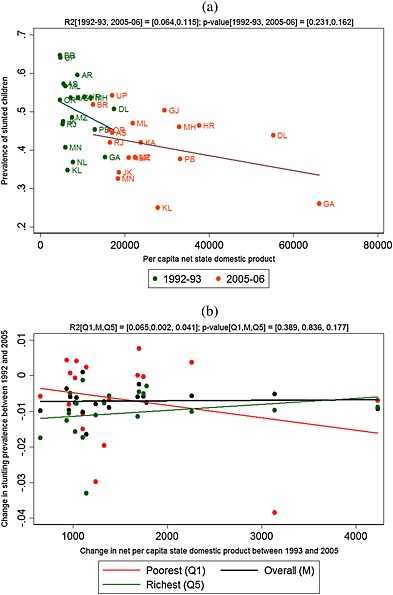
Association of (a) state‐level economic growth and state‐level prevalence of stunting in India, and (b) change in state‐level economic growth and change in state‐level prevalence of stunting for children from the poorest and richest quintile of household wealth as well as all children, between 1992–1993 and 2005‐2006. (*) Change in stunting prevalence = (prevalence in 2005–2006 − prevalence in 1992–1993) / 13. Change in net per capita domestic product = (state product in 2005–2006 − state product in 1992–1993) / 12. Data for stunting are from National Family Health Survey (NFHS)‐1 and NFHS‐3. Data for per capita net state domestic product (PCNSDP) are from Subramanyam *et al*. (2011).

**Figure 5:**
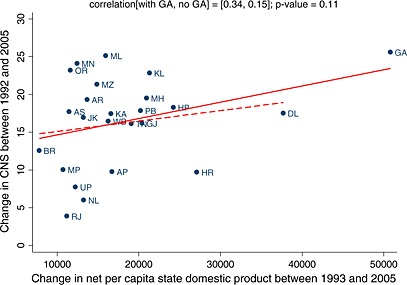
Association between change in state‐level economic growth and change in state‐level Child Nutrition Score (CNS). Source: Authors' calculations using data from National Family Health Survey (NFHS)‐1 and NFHS‐3. (*) The dashed line was estimated after excluding GA. State abbreviations: AS, Assam; BR, Bihar; GA, Goa; GJ, Gujarat; HR, Haryana; JK, Jammu and Kashmir; KA, Karnataka; KL, Kerala; MH, Maharashtra; MN, Manipur; ML, Meghalaya; MZ, Mizoram; NL, Nagaland; OR, Odisha; PB, Punjab; RJ, Rajasthan; UP, Uttar Pradesh; DL, New Delhi; AR, Arunachal Pradesh. Child Nutrition Score (CNS) – We followed Aguayo *et al*. (2014) to estimate a CNS using five risk factors constructed using NFHS‐1 and NFHS‐3. The risk factors included were as follows: (1) early initiation of breastfeeding; (2) exclusive breastfeeding under 6 months; (3) timely introduction of complementary foods; (4) full vaccination; and (5) access to improved sanitary facilities. Although Aguayo *et al.* (2014) recommend the use of 10 proven essential interventions in the construction of CNS, we only used the first four indicators that we were able to estimate consistently from the available information in NFHS‐1 and NFHS‐3 and used *access to improved sanitary facilities* as a proxy for *safe disposal of stools*.

Finally, income generation (even when it happens) cannot immunize from the broader ‘supply side’ deficiencies. Even if it were the case that economic growth did lead to substantial improvements in individuals' incomes, and especially those of the poor, this would still impact only few of the proximal risk factors that can causally reduce undernutrition, e.g. access to sufficient food. Even here, proper infrastructure (e.g. Public Distribution Systems or well‐regulated markets) would have to be in place to efficiently deliver food at affordable prices. In India, where food inflation has been rampant, there is hardly any evidence that income improvements have vastly outstripped the food inflation, especially for the poor (Sen 2008). In fact, the evidence suggests a decline in calorie consumption in India (Deaton & Drèze 2009).

More importantly, reductions in child undernutrition are also dependent on other risk factors that are unlikely to automatically improve as direct consequences of higher household incomes. These include access to clean water and sanitation, as well as access to treatment to reduce recurring morbidities and prevention of infection through immunization. Improvements in these conditions are influenced by robust public investments, which often depend more on the policy and political environment than on the availability of resources. In countries such as India, there is no compelling evidence that economic growth has led to improved access to clean water and sanitation or treatment. For instance, as surprising as this might be for a casual observer, India has been formally declared to have achieved the Millennium Development Goal 7c related to water, which is to halve the proportion of the population without sustainable access to safe drinking water and basic sanitation by 2015 (WHO/UNICEF 2013). However, a recent study found alarmingly high contamination rates of water from the so‐called ‘improved’ water sources in India (Johri *et al.* 2014). Although nearly 100% of the surveyed households in both urban and rural areas had access to ‘safe water’, as per the Millennium Development Goal definition, water tests revealed contamination in approximately 40% and 60% of urban and rural households, respectively (Johri *et al.* 2014).

In summary, before advocates of growth‐mediated strategy extol the role of macroeconomic growth as a policy instrument to reduce child undernutrition, it is critical to recognize the aetiology of undernutrition and mechanistic connections that are required to generate associations between growth and undernutrition. As the connections are far from being direct, it is critical to explore why undernutrition exists and is more prevalent among poorer individuals and how economic growth can improve nutritional status. Indeed, if economic growth is actually ‘pro‐poor’, and the increased public revenue as a consequence of such growth is invested in improving the causative agents – structural as well as proximal – which matter for child undernutrition, then growth, of course, can have an impact on undernutrition. Sadly, the evidence and reality appear to be the contrary.

## The folly of framing undernutrition as a ‘demand‐side’ problem

In addition to viewing economic growth as a panacea, the scientific and policy discourse tends to view undernutrition as a ‘demand‐side’ problem. Under such a diagnosis, invariably, resources and efforts are disproportionately shifted to strategies for promoting behaviour change among individuals rather than investing in structural interventions. Such demand‐side framing often triggers ‘downstream’ interventions that address the symptoms of the cause (‘proximal risk factors’) as opposed to the structural causes that are usually ‘supply‐side’. To illustrate the folly of demand‐side approach consider the current focus on, as well as efforts to address, the problem of open defecation in India, viewed as one of the major explanation for why Indian children are shorter (Harris 2014). Why do Indians defecate in the open?

One explanation, which is partially targeted at infrastructural aspects, might be because there is no provision for defecating in an enclosed and dedicated space (i.e. a latrine) in households. For instance, 69% of the rural households have no latrines in their homes, whatsoever; and only 19% of the households have ‘improved’ latrines (water closet) (Census of India 2011). Further, there are also large socioeconomic inequities in the quantity and quality of latrines (Table 2). It will not be an exaggeration to state that a water‐based latrine (i.e. whereby excreta is transported with water or the ‘flush latrine’) is the ‘norm’ for the higher socioeconomic groups and perhaps the ‘aspiration’ for those who do not possess this luxury. A clear gradient is observed across socioeconomic groups for improved facilities; for example, nearly 90% of households in the highest wealth quartile have access to flush toilets connected to the piped sewer system, while it is 0% for those in the lowest wealth quartile (Table 2a). Conversely, lowest wealth quartile households either have no latrine (42%), compared with 2% for the highest wealth quartile households; and when they do, they are largely of the ‘not improved’ category of latrines (e.g. pit latrine without slab or open pit or a dry latrine) (Table 2a). Moreover, nearly 75% of households in the highest wealth quartile have access to an improved toilet facility, whereas nearly all households in the lowest wealth quartile have no latrine (95%) (Table 2b).

**Table 2:** Weighted percentage of households with access to sanitary facility (a) by wealth quartile and (b) by type of facility in every wealth‐quartile group in India, 2005–2006

Type of facility	Wealth quartile (%)			
	1	2	3	4
(a)				
Improved				
Flush to piped sewer system	0	0.7	10.4	88.9
Flush to septic tank	0.3	4.3	27.7	67.6
Flush to pit latrine	3.4	19.7	39	37.8
Ventilated improved pit latrine (vip)	1.4	12.9	28.5	57.3
Pit latrine with slab	12.1	28.7	35.6	23.6
Composting toilet	0.7	21.6	44.2	33.5
Not improved				
Flush to somewhere else	0.6	4.5	24.5	70.3
Flush, don't know where	2.4	4.2	49.7	43.7
Pit latrine without slab/open pit	22.1	46.3	26.7	4.8
No facility/bush/field	42.7	36.2	19.2	1.9
Dry toilet	20.6	43.2	29.5	6.7
Other or shared	4.6	15.9	47	32.5
(b)				
Improved				
Flush to piped sewer system	0	0.2	2.7	23.4
Flush to septic tank	0.2	2.8	17.9	43.7
Flush to pit latrine	0.6	3.4	6.7	6.5
Ventilated improved pit latrine (vip)	0	0.1	0.2	0.4
Pit latrine with slab	0.9	2.2	2.7	1.8
Composting toilet	0	0	0.1	0
Not improved				
Flush to somewhere else	0	0.2	0.9	2.6
Flush, don't know where	0	0	0.1	0.1
Pit latrine without slab/open pit	1	2.1	1.2	0.2
No facility/bush/field	94.5	80.1	42.5	4.3
Dry toilet	0.3	0.6	0.4	0.1
Other or shared	2.4	8.3	24.5	16.9

*Source:* Author calculations using data from NFHS‐3 (IIPS 2007).

Another explanation, which appears to gain considerable currency in the policy and scientific discourse, is that open defecation is a demand‐side problem, i.e. Indians *prefer* defecation in the open for various behavioural, cultural and religious reasons (Harris 2014; Coffey *et al.* 2015). Ignoring the stark realities and inequities outlined earlier with regard to dramatic shortfalls and inequities in quantity and quality, arguments have been made that even when latrines are available, they are not used (Barnard *et al.* 2013; Coffey *et al.* 2014). It is hard to resist wondering, if cultural reasons dominate over availability of an improved quality latrine, whether migrant Indians (poor or rich) practice open defecation in the richer countries to which they often emigrate? We are not aware of any documented evidence for such a practice.

Meanwhile, current efforts to end open defecation in India have focused on two things: (1) behaviour change (an intervention that presumes that open defecation is a demand‐side problem) and (2) behaviour change by building a latrine in the home (a demand‐side presumption along with a proximal intervention). Two recent randomized trials on toilet construction and awareness‐raising projects studied whether such changes reduce the incidence of diarrhoeal disease or stunting (Clasen *et al.* 2014; Patil *et al.* 2014). The first trial, conducted in 80 rural villages in Madhya Pradesh, included promotion and subsidy of improved sanitation facilities as well as educational campaigns to change defecation behaviours and child health (Patil *et al.* 2014). Despite slight increases in the percentage of household with improved latrines and modest reductions in the open defection practices, the intervention was ineffective in reducing the prevalence of gastrointestinal illness, intestinal parasite infections and increasing growth in children under 5 years of age (Patil *et al.* 2014). The second randomized experiment, conducted in 100 rural villages in Odisha, which involved installation of improved sanitation facilities, revealed no reduction in the exposure to faecal contamination or prevented diarrhoea, soil‐transmitted helminth infections or child malnutrition (Clasen *et al.* 2014).

We hope that such thoughtless experiments will not discourage from focusing on the aspects of water and sanitation, which is clearly in a state of crises, as a means for addressing the problem of undernutrition. They are thoughtless because we *do* know how to dramatically bring about improvements in child health through improvements in water and sanitation. It is well documented that substantial improvements in reducing child mortality and improvements in child health in the early 19th century in industrialized countries were linked to substantial investments in supply*‐*side public health infrastructure related to public hygiene (Cutler & Miller 2005; Semba 2008). Yet demand*‐*side interventions are ubiquitous when it comes to addressing the most basic and fundamental needs of living a dignified human life in low‐income countries.

Open defecation in India is a symptomatic reflection of the abject failure at the structural level, which is both inadequate and highly inequitable (Narain 2002, 2012). The issue, as it relates particularly to undernutrition, is how should human excreta be disposed of and handled. Of the total excreta from the ‘*closed* defecations’ (i.e. the type of defecation practiced by upper‐income groups), 80% is returned back to the grounds, rivers and seas completely untreated (Narain 2002). It is worth bringing a political economy perspective to treating human excreta in India; as Narain (2002) shows sewer systems in India are subsidized for the use of the better‐off groups in cities, while the poor (especially those in rural areas) left with no latrines or to experiment with alternate (‘ecosanitation’) types of latrines that are nowhere close to the ‘flushed latrines connected to a closed sewer’. Such ecosanitation technologies are likely to be interim alternatives when proposed for the unserved poor and most likely discarded for the ‘aspirational’ latrine when people's incomes increase (Narain, 2002). Furthermore, as much as the excreta from open defecation can be an environmental risk factor for undernutrition, the excreta from closed defecation, while remains out of sight of the homes, equally poses the same magnitude of environmental risk, given the lack of integrated infrastructure to treat the excreta before it is back into our environments. It is also important to recognize that India on a classification of ‘water stress’ (ratio of withdrawals to supply) levels is considered ‘high stress’ (Reig *et al.* 2013), and a thoughtful debate and discussion on the appropriate and equitable technology suited for India that links water and sanitation is woefully missing. In absence of discussions and efforts focused on structural, supply‐side conditions, the proposal to construct 110 million toilets (PIB/GOI 2015), while it will attenuate the socioeconomic inequalities with regard to access to latrines (even if of the unimproved type) may not have a considerable impact on reducing undernutrition.

While we use open defecation to exemplify the over‐reliance on the demand‐side approaches to intervene on child undernutrition, there are other examples as well. For instance, in India, the Expert Task Force on Infant and Young Child Nutrition of the Coalition for Sustainable Nutrition Security identified 10 strategies to address the high levels of undernutrition, all of which address only nutrition‐specific, demand‐side determinants such as breastfeeding, introduction of complementary foods and feeding practices (Box 1) (Swaminathan 2009). In addition, many programmes focus on promoting breastfeeding through both individual and group counselling (Haroon *et al.* 2013). Other programmes encourage parents to provide complementary foods (Lassi *et al.* 2013) or vitamin supplementation to their children (see studies in Bhutta *et al.* 2013). Priorities and programmes that only promote behaviour change ignore other drivers of undernutrition such as household poverty that may prevent families from affording high‐quality foods or vitamin supplements or may affect women's ability to breastfeed (Black *et al*. 2013; Ruel *et al*. 2013). Even though there is broad consensus that commensurate investments in supply‐side, nutrition‐sensitive programmes such as improvements in agricultural sectors and strengthening of social welfare programmes, as well as broader investments in early child development and schooling are required to substantially reduce undernutrition (Bhutta *et al.* 2008; Bhutta *et al.* 2013; Gillespie *et al* 2013; Ruel *et al*. 2013, Pinstrup‐Anderson, 2013); in practice, there is little evidence of such complementary approaches.

**Table Box 1:** Ten priority areas

1. Timely initiation of breastfeeding within 1 h of birth
2. Exclusive breastfeeding within the first 6 months of life
3. Timely introduction of complementary foods at 6 months of age
4. Age‐appropriate complementary foods, adequate in terms of quality, quantity and frequency for children 6–23 months
5. Safe handling of complementary foods and hygienic complementary feeding practices
6. Full immunization and bi‐annual vitamin A supplementation with deworming
7. Frequent, appropriate and active feeding for children during and after illness, including oral rehydration with zinc supplementation during diarrhoea
8. Timely and quality therapeutic feeding and care for all children with severe acute malnutrition
9. Improved food and nutrient intake for adolescent girls particularly to prevent anaemia:
10. Improved food and nutrient intake for women, including during pregnancy and lactation

Source: Swaminathan *et al.* 2009.

In developed countries, supply‐side structural interventions targeting social, economic, political and environmental determinants have had significant impact on improving child health and nutrition. Specific examples of such interventions include water filtration and chlorination, which provide clean, drinkable water and reduce exposure to water‐borne illnesses. In response to the high incidence of cholera and other disease spread by drinking water from the Thames, the Metropolis Water Act of 1842 was enacted, decreeing that water be drawn from only particular regions of the river and be filtered before public distribution (Semba 2008). In the United States, nearly three quarters of reductions in infant mortality and half child mortality were attributable to introduction of filtration and chlorination in the early 20th century (Cutler & Miller 2005). Other examples of structural interventions include new institutions and laws. The British Public Health Act of 1848 established a general board of health and enacted legislation to oversee food production and processing, water and sanitation with similar public health measures enacted in the United States (Semba 2008). In the early 1900s, much attention was also paid to the high infant mortality rates with promotion of breastfeeding, maternal education, better prenatal care and pasteurization of milk (Semba 2008). Other factors contributing to better nutritional outcomes include advanced agricultural technologies and relatedly increases in food production (Semba 2008). There were also improvements in food distribution systems, namely, the construction of railways (Semba 2008). Developing countries would do well to take lessons learned from these advances in developed countries and invest more in structural interventions.

## Shifting from a single, siloed risk factor to an integrated multifactorial approach

Alongside a disproportionate focus on demand‐side approaches to policy action, there is a tendency to consider one proximal risk factor at a time (often in an independent manner), as opposed to considering the simultaneous influence of different risk factors and appreciating the linkages between the different risk factors. The focus on single risk factors is partially justified by how effectiveness studies are designed. Most programme evaluations marshal evidence from randomized control trials or quasi‐experimental designs (Bhutta *et al*. 2008; Bhutta *et al*. 2013), which only manipulate a single factor to assess its effect. However, there is a call to integrate evidence from these disparate studies into multisectoral interventions (Casanovas *et al*. 2013; de Onis *et al*. 2013; Ruel *et al*. 2013). Linked interventions are supported by studies from Brazil and sub‐Saharan Africa that demonstrate that integrated interventions have led to substantial reductions in stunting (Casanovas *et al*. 2013). In parallel, there is a need to better evaluate multisectoral interventions to support further investment (Ruel *et al.* 2013).

The broader dilemma of a single factorial vs. multifactorial is perhaps tellingly captured in the parable by the medical sociologist, Irving Zola (Box 2). In the parable, an onlooker witnesses a man caught in a river current. The onlooker saves the man by pulling the man out of the river, resuscitating him and providing first aid, only to be drawn to the rescue of another drowning person, with the same cycle – pulling, resuscitating and treating – continuing. Caught up in the frenzy of efforts to rescue the drowning man, one forgets to ask: what is happening ‘upstream’ that is triggering the fall of so many people into the river? The ‘emergency’ nature of responding to child undernutrition with one risk factor at a time, as important and relevant as they are, only offer the ‘first aid’, and therefore, likely to be short term in nature (McKinlay 1979). In the absence of a simultaneous attention to the upstream conditions (i.e. the root causes and origin of the problems and often multifactorial in nature), single‐factorial approaches might even be entirely futile (McKinlay 1979). This fundamental idea also forms the basis for the report of the World Health Organization (WHO) Commission on Social Determinants of Health (WHO 2008). Indeed, the theoretical and conceptual frameworks for addressing undernutrition are quite rich and capture the *multilevel* determinants of undernutrition (Black *et al.* 2008; Black *et al.* 2013; UNICEF 2013). Yet the practice (both in terms of scientific research as well as policy interventions) is often incredibly myopic to the underlying conceptual framework.

**Table Box 2:** The Parable by Irving Zola

‘You know’, he said, ‘sometimes it feels like this. There I am standing by the shore of a swiftly flowing river and I hear the cry of a drowning man. So I jump into the river, put my arms around him, pull him to shore and apply artificial respiration. Just when he begins to breathe, there is another cry for help. So I jump into the river, reach him, pull him to shore, apply artificial respiration, and then just as he begins to breathe, another cry for help. So back in the river again, reaching, pulling, applying, breathing and then another yell. Again and again, without end, goes the sequence. You know, I am so busy jumping in, pulling them to shore, applying artificial respiration, that I have no time to see who the hell is upstream pushing them all in’.

Reproduced from McKinlay (1979).

The need for integrated and multifactorial thinking, as opposed to addressing one risk factor at a time cannot be emphasized enough. A recent study examining simultaneously the relative influence of vitamin A supplementation, vaccination, use of iodized salt, household air quality, improved sanitary facilities, safe disposal of stools, improved drinking water, prevalence of infectious disease, initiation of breastfeeding and dietary diversity (all identified as ‘risk factors’ for undernutrition) and broader social determinants such as age of marriage, maternal body mass index, height, education and household wealth found that these social determinants had a combined population attributable risk of 63.5% for stunting (Corsi *et al*. 2015). To reduce the prevalence of undernutrition, it is imperative to jointly address the multiple determinants operating at various levels. Indeed, the recent failures of randomized trials of Vitamin A supplementation for child survival (Haider & Bhutta 2015) further underscore the limits of a single‐factorial approach to improving child health.

Studies examining the effectiveness of these single risk factor approaches reveal the shortcomings of these programmes. Not surprisingly, effects from interventions are very small. For example, multiple micronutrient supplementation in children ages 6 months to 16 years led to a 0.13‐cm increase in length, while zinc supplementation for 24 weeks led to 0.37‐cm increase in height among children younger than age 5 years (Bhutta *et al.* 2013 and Table 3). Furthermore, if these interventions were to be scaled up from experimental studies to the population level, 90% coverage would lead to only a 20% reduction in stunting (Bhutta *et al.* 2013). The greatest reductions in undernutrition would be brought about by approaches that target the multiple dimensions of undernutrition, linking behaviour change programmes with interventions to address the structural determinants (Corsi *et al*. 2015).

**Table 3:** Demand‐side interventions

Intervention	Estimates
Breastfeeding promotion	Effects of educational/counselling interventions on the following: *Early breastfeeding*: By 43% (95% CI: 9–87) on day 1, by 30% (95% CI: 19–42) at 1 month, and by 90% (95% CI: 54–134) from 1–6 months.
	*No breastfeeding*: By 32% (95% CI: 13–46) at day 1, 30% (95% CI: 20–38) at 0–1 month, and 18% (95% CI” 11–23) for 1–6 months.
Complementary feeding promotion (children ages 6–24 months)	*Nutrition education in food secure populations*: Height gain (standardized mean difference = 0.35; 95% CI 0.08–0.62), HAZ (standardized mean difference = 0.22; 95% CI 0.01–0.43).
	No significant effects on stunting
	*Nutrition education in food insecure populations*: HAZ (standardized mean difference 0.25, 95% CI 0.09–0.42), stunting (relative risk: 0.68, 95% CI 0.60–0.76),
	*Provision of complementary food with and without education in food insecure populations*: HAZ (standardized mean difference = 0.39, 95% CI 0.05–0.73)
Multiple micronutrient supplementation	Children ages 6 months to 16 years receiving supplementation had 0.13 cm (95% CI 0.06–0.21) greater length
Zinc supplementation	Children under age 5 years receiving supplementation for 24 weeks experienced 0.37‐cm (0.25‐SD) increases in height, on average
WASH interventions	20 percentage point reduction in open defecation was associated with 0.1‐SD increase in child height

CI, confidence interval; HAZ, height‐for‐age *z*‐scores; SD, standard deviation.

Source: Bhutta *et al*. 2013.

## Concluding remarks

In their book, *Hunger and Public Action*, Drèze and Sen (1991) make the distinction between pursuing a ‘growth‐mediated’ and the ‘support‐led’ strategy to address the problem of nutritional deprivation. The ‘growth‐mediated’ strategy relies on promoting economic growth leading to greater employment opportunities and related increases in household income. The increasing affluence could also indirectly provide the basis for inducing demand for better public services (e.g. increased public expenditure to ensure adequate and quality of relevant services) that are critical for improving nutritional status. In contrast, the ‘support‐led’ strategy involves direct interventions in creating equitable public policies and provisions that matter for nutrition (e.g. ensuring a strong food distribution system or investments in water and sanitation infrastructure).

Importantly, the two strategies are not mutually exclusive and are likely to be synergistic. There is also considerable evidence that investing in health and nutrition, especially during childhood, has substantial economic pay‐offs. It turns out that in ‘cost‐benefit’ analyses of investing in child nutritional programmes, the benefits (both short and long terms) substantially outweigh the costs of designing and implementing interventions. For instance, for every dollar spent on child nutrition programmes, the returns were valued at $34 for India (Deolalikar 1988). Indeed, with 30% of India's population under the age of 15 years (United Nations 2013), it is imperative to invest in the overall child development if India is to reap its ‘demographic dividend’ (Bloom *et al.* 2003). It is the quality (healthy and educated) of the younger population, and not just the quantity, that will decide the extent to which any such dividends India might be able to accrue.

The evidence that a growth‐mediated strategy can improve nutritional status among children is no longer compelling. Advocates for a growth‐mediated strategy will continue to argue that economic growth has to be considerably higher and more rapid than what India has experienced, or that economic growth can be engineered to be substantially more broad‐based, and that it is not advisable to dismiss the role of economic growth in improving nutritional status of children in India. Notwithstanding the merits of such an argument, there is no reason (moral or economic) to delay pursuing an equally aggressive support‐led strategy focused on supply‐side structural interventions with a multifactorial approach to substantially reducing the incidence of childhood stunting, as well as improving the lives of millions of children in South Asia.

## Source of Funding

None.

## Contributions

SVS conceptualized and wrote the article. IMG contributed to the data analysis and writing. AK contributed to the literature review and writing. Work done by AK was performed while a doctoral student at the Harvard T. H. Chan School of Public Health.

## Conflicts of interest

The authors declare that they have no conflicts of interest.

## Supporting information

Supporting info item
