# Supplementary material for: Rethinking policy perspectives on childhood stunting: time to formulate a structural and multifactorial strategy
Source: Matern Child Nutr. 2016 May 17;12(Suppl 1):219–36. doi: 10.1111/mcn.12254 (PMC5084745; doi:10.1111/mcn.12254)
Supplement: Supplementary file 1 — Supporting info item [file MCN-12-219-s001.docx]

**Supplementary Material**

**Methods**

Data for this study came from the cross-sectional and nationally representative Indian National Family Health Surveys from 1992-93 (NFHS-1) and 2005-06 (NFHS-3). NFHS is part of the Demographic Health Survey (DHS) Program, which had conducted similar surveys in more than 90 low-to-middle income countries (MEASURE DHS 2015). NFHS provides indicators of population, nutrition, and health with primary focus on women in reproductive ages (15-49) and their under-five age children.

NFHS-1 was based on a stratified multi-stage cluster survey design. Stratification was conducted by rural and urban location and by state. In the rural sample, a two-stage sampling design was adopted, where villages where drawn in the first stage followed by households at the second stage. Villages were further stratified before selection based on population and geophysical characteristics to delineate Primary Sampling Units (PUSs), which were systematically selected with probability Proportional to Population Size (PPS). In in the second sampling stage, an average of 30 previously listed households was selected systematically with equal probability within each selected PSU. In the urban sample, a three-stage sampling design was adopted. Cities/towns were drawn in the first stage with PPS, followed by urban blocks (PSUs) in the selected towns in the second stage, and by the selection of an average of 20 households within each selected block with equal systematic probability. As in the rural case, all households were listed within each selected PSU (IIPS 1995). The sampling design and selection in the NFHS-3 was similar, with only subtle differences (IIPS and Macro International 2007).

Children less than five years old were initially considered for this study. However, we restricted analyses to children under age 4 years, because the NFHS-1 only surveyed individuals in that age range. The initial sample was composed of 90,009 children, but 5,887 (6.5%) dead children and 953 (1.1%) who were born in multiple births were excluded. In addition, 10,855 children were also excluded because were not measured [9435 (10.5%)] or with missing observations [710 (0.8%)]. Finally, we further dropped information from 11,469 children with Height-for-age (HAZ) measures below -6 or above 6 SD of the median reference population, defined by the World Health Organization (WHO) as biologically implausible (WHO 2006b). The final analytic sample then included 60,845 children.

Length/height was measured by trained personnel to children under/above 2 years of age old using adjustable boards accurate to the nearest millimeter. To measure the nutritional status we used the Height-for-age z-score (HAZ), which is a standard index defined by the WHO based on reference population of children the same age and sex (WHO 2006a; WHO 2006b). We defined stunted or short by their age children as those whose HAZ z-score were below -2 SD of the median of the WHO reference population. We used ©Stata software *zscore06* to estimate these anthropometric measures (Leroy 2011). We retrieved wealth indexes available in NFHS based on possession of household assets, which were used to score households and rank individuals in wealth quintiles according to the rank of the household where they reside. We estimated a modified version of the wealth index by following the methodology applied to the index provided in the NFHS (Rutstein & Johnson 2004), but excluding drinking water sources and toilet facilities, and ranked individuals in wealth quartiles using this index. Wealth quintiles where used for most analyses, with exception for the prevalence analysis of type of toilet facilities, where we applied wealth quartiles.

**Change in stunting prevalence in the richest wealth quintiles (fourth and fifth)**

Using information from NFHS-1 and NFHS-3 and Census data from India in 1991 and 2001, we estimated the number of children under age 5 in each wealth quintile group by Indian state, and then multiplied the number of these children by the state per capita GDP to approximate the state-level domestic product by wealth quintile group, attributable to that specific age group. We then focused the analysis on the richest groups (quintiles fourth and fifth) to investigate the association between changes in state-level stunting prevalence between the period 1992 and 2005 changes in state-level economic growth in these specific groups. Even though we found an significant ecological association between levels of stunting and the state level domestic product using 2005 data [**Figure A1(a)** and **(b)**], no significant association was found when analyzing the change in economic growth and the change in stunting prevalence in any socioeconomic subgroup [**Figure A1(c)**]. A limitation of this approach is that we were able to get only a rough approximation of the state-level domestic product by quintile group since, to the best of our knowledge, that information is not available in India.

**Figure A1.** Association of state-level economic growth (in log scale) and state-level prevalence of stunting in India, for quintiles (a) fourth (Q4) and (b) fifth (Q5) of household wealth, and (c) change in state-level economic growth (in log scale) and change in state-level prevalence of stunting for children in Q4 and Q5, between 1992-93 and 2005-06.

| 1. Quintile 4   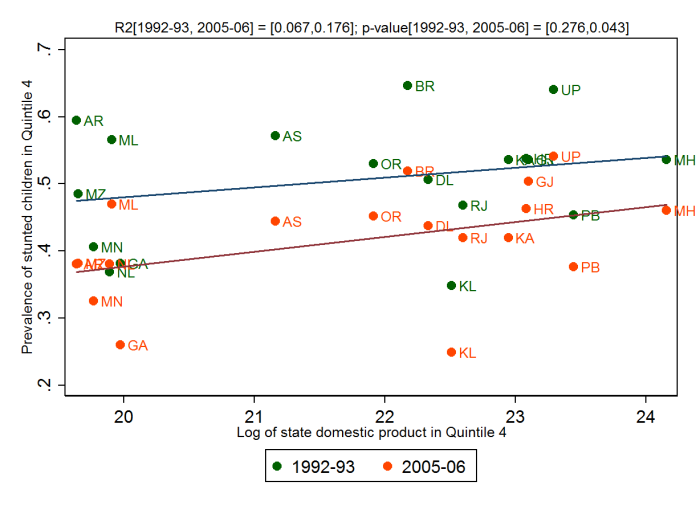 | 1. Quintile 5   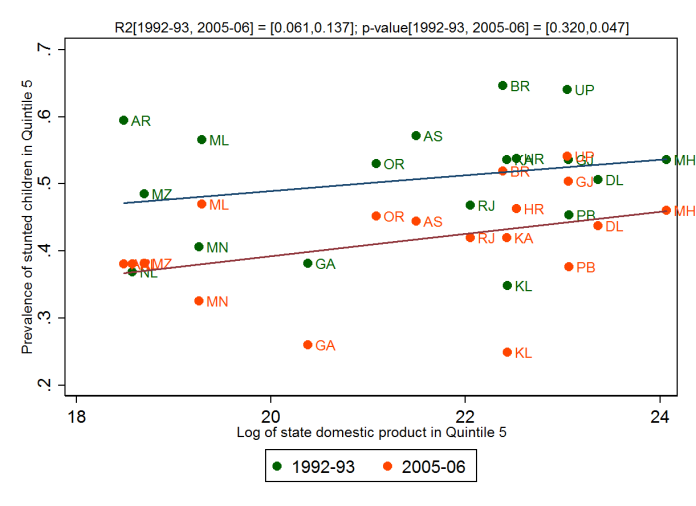 |
| --- | --- |
| 1. Change: Q4 and Q5   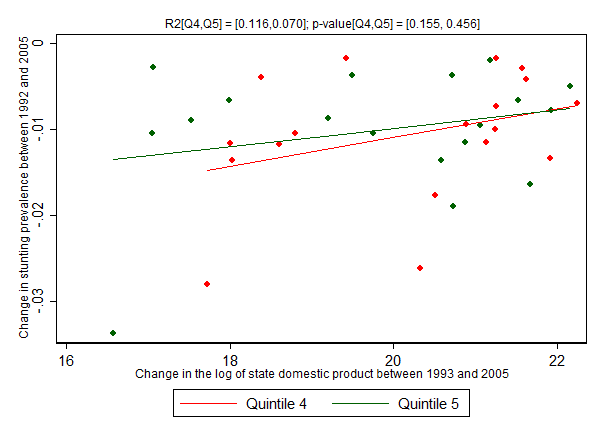 | |

**Supplementary References**

1. International Institute for Population Sciences (IIPS) (1995) National Family Health Survey (MCH and Family Planning), India 1992-93. Bombay: IIPS.
2. International Institute for Population Sciences (IIPS) and Macro International (2007) National Family Health Survey (NFHS-3), 2005–06: India: Volume II. Mumbai: IIPS.
3. Leroy J. L. (2011). ‘zscore06’: Stata command for the calculation of anthropometric z-scores using the 2006 WHO child growth standards. Retrieved 1 February 2015 from <http://www.ifpri.org/staffprofile/jef-leroy>.
4. MEASURE DHS. All Surveys by Country. http://dhsprogram.com/What-We-Do/Survey-Types/DHS.cfm (31 August 2015, date last accessed).
5. Rutstein S.O., Johnson K. (2004) The DHS Wealth Index. DHS Comparative Reports No. 6. Calverton, MD: ORC Macro.
6. WHO Multicentre Growth Reference Study Group (2006a) Assessment of differences in linear growth among populations in the WHO Multicentre Growth Reference Study. Acta Paediatrica **450**, 56–65.
7. World Health Organization (WHO) (2006b) WHO Child Growth Standards: Length/Height-for-Age, Weight-for-Age, Weight-for-Length, Weight-for-Height and Body Mass Index-for-Age: Methods and Development. Geneva, Switzerland: World Health Organization.

**State and Country Labels for Fig. 3**.

***State abbreviations:*** AP, Andhra Pradesh; AR, Arunachal Pradesh; AR, Arunachal Pradesh; AS, Assam; BR, Bihar; CH, Chhattisgarh; GA, Goa; GJ, Gujarat; HR, Haryana; HP, Himachal Pradesh; JK, Jammu and Kashmir; JH, Jharkhand; KA, Karnataka; KL, Kerala; MP, Madhya Pradesh; MH, Maharashtra; MN, Manipur; ML, Meghalaya; MZ, Mizoram; NL, Nagaland; DL, New Delhi; OR, Odisha; PB, Punjab; RJ, Rajasthan; SK, Sikkim; TN, Tamil Nadu; TR, Tripura; UP, Uttar Pradesh; WB, West Bengal.

***Country abbreviations:*** AL: "Albania, 2008"; AZ: "Azerbaijan, 2006"; BA: "Bangladesh, 2011"; BE: "Benin, 2011"; BO: "Bolivia, 2008"; BF: "Burkina Faso, 2010"; BU: "Burundi, 2010"; CA: "Cambodia, 2010"; CR: "Cameroon, 2011"; CH: "Chad, 2004"; CO: "Colombia, 2009"; CM: "Comoros, 2012"; CD: "Congo, Democratic Republic, 2013"; CR: "Congo, Republic, 2011"; CI: "Cote d'Ivoire, 2011"; DR: "Dominican Republic, 2013"; ET: "East Timor, 2009"; EG: "Egypt, 2008"; ET: "Ethiopia, 2010"; GA: "Gabon, 2012"; GH: "Ghana, 2008"; GU: "Guinea, 2012"; GY: "Guyana, 2009"; HA: "Haiti, 2012"; HO: "Honduras, 2011"; IN: "India, 2005"; JO: "Jordan, 2012"; KA: "Kazakhstan, 1999"; KE: "Kenya, 2008"; KR: "Kyrgyz Republic, 2012"; LE: "Lesotho, 2009"; LI: "Liberia, 2013"; MA: "Malawi, 2010"; MD: "Maldives, 2009"; ML: "Mali, 2012"; MO: "Moldova, 2005"; MR: "Morocco, 2003"; MZ: "Mozambique, 2011"; NA: "Namibia, 2013"; NE: "Nepal, 2011"; NI: "Nicaragua, 2001"; NG: "Niger, 2012"; NR: "Nigeria, 2013"; PA: "Pakistan, 2012"; PE: "Peru, 2012"; RW: "Rwanda, 2010"; ST: "Sao Tome and Principe, 2008"; SL: "Sierra Leone, 2013"; SW: "Swaziland, 2006"; TJ: "Tajikistan, 2012"; TA: "Tanzania, 2009"; TO: "Togo, 2013"; TU: "Turkey, 2003"; UG: "Uganda, 2011"; ZA: "Zambia, 2013"; ZI: "Zimbabwe, 2010".
